# Contribution of Different Positron Emission Tomography Tracers in Glioma Management: Focus on Glioblastoma

**DOI:** 10.3389/fonc.2019.01134

**Published:** 2019-11-01

**Authors:** Aurélie Moreau, Olivia Febvey, Thomas Mognetti, Didier Frappaz, David Kryza

**Affiliations:** ^1^Centre Léon Bérard, Lyon, France; ^2^UNIV Lyon - Université Claude Bernard Lyon 1, LAGEPP UMR 5007 CNRS Villeurbanne, Villeurbanne, France; ^3^Hospices Civils de Lyon, Lyon, France

**Keywords:** glioblastoma, imaging, PET, FDG, DOPA, radiolabeled amino acids, PSMA

## Abstract

Although rare, glioblastomas account for the majority of primary brain lesions, with a dreadful prognosis. Magnetic resonance imaging (MRI) is currently the imaging method providing the higher resolution. However, it does not always succeed in distinguishing recurrences from non-specific temozolomide, have been shown to improve -related changes caused by the combination of radiotherapy, chemotherapy, and targeted therapy, also called pseudoprogression. Strenuous attempts to overcome this issue is highly required for these patients with a short life expectancy for both ethical and economic reasons. Additional reliable information may be obtained from positron emission tomography (PET) imaging. The development of this technique, along with the emerging of new classes of tracers, can help in the diagnosis, prognosis, and assessment of therapies. We reviewed the current data about the commonly used tracers, such as 18F-fluorodeoxyglucose (18F-FDG) and radiolabeled amino acids, as well as different PET tracers recently investigated, to report their strengths, limitations, and relevance in glioblastoma management.

## Introduction

Gliomas are the most common primary malignant brain tumors arising from glial cells. The glial cells form the neuronal environment and are involved in myelin production and in the support and protection of the neurons. A new World Health Organization (WHO) classification has been proposed recently, combining the gliomas both from histological and molecular findings and classifying them into four grades (1). Glioblastomas are the most frequent type of gliomas in adults (about 55%), with an increasing annual incidence of 3–4 per 100,000 people newly diagnosed in the USA and Europe. The disease is slightly more frequent in males than in females. They are particularly locally aggressive, with rare cases of metastases. The prognosis is dismal with a 5-year survival rate of lower than 10% (2, 3). According to the 2016 WHO classification, two main subtypes of glioblastomas are identified: the isocitrate dehydrogenase (IDH)-wildtype and the IDH-mutant variants. The IDH mutation leads to a D2-hydroxyglutamate overproduction, involved in a large number of cellular reactions and in histone and DNA hypermethylation (4, 5). Primary or *de novo* glioblastoma is frequently characterized by the presence of the IDH-wildtype isoform in ~90% of cases, occurring in older patients. The other 10% with the IDH-mutant variant is seen in younger patients with secondary glioblastoma, with a prior history of lower grade diffuse glioma (1). The risk factors currently identified are the exposure to therapeutic doses of radiation and genetic syndromes (such as neurofibromatosis 1 and 2 and the Li-Fraumeni syndrome) (6). Clinical symptoms include headaches, epileptic seizures, focal neurologic deficit, confusion, memory loss, and personality modifications, depending on the location of the tumor (7). The gold standard treatment is surgical resection, radiotherapy, and chemotherapy (8, 9). Although complete surgical resection of glioblastomas is often not achievable due to their highly infiltrating nature, the extent of surgical resection remains a key component for survival improvement (10–12). The second cornerstone is subsequent radiation therapy that can lead to an improvement in survival rate of 6 months (13). For patients up to 71 years, the standard treatment is adjuvant administration of temozolomide chemotherapy treatment, leading to an improvement in progression-free survival (PFS) and overall survival (OS) (14). All patients with a 2.5 months global survival benefit are eligible (15). However, a better efficacy is observed in patients with a methylated MGMT promoter (3, 16). Targeted therapies, such as the anti-VEGF agent bevacizumab in association with temozolomide, have been shown to improve PFS, but no impact on OS has been reported (17, 18). Despite the initiation of such aggressive treatments, relapses are the rule. The reference imaging technique to monitor the onset of recurrences is magnetic resonance imaging (MRI), more specifically, multimodal MRI (MRI with gadolinium injection associated with spectroscopy, perfusion, and diffusion). Glioblastomas conventionally appear as hypo or iso-intense on T1, enhanced in “a ring pattern” in T1 with gadolinium, and are hyper-intense on T2 and FLAIR acquisitions. The challenge is to improve diagnosis and to discriminate post-therapeutic recurrences from radiation complications, such as pseudoprogression or radiation necrosis, and from pseudoresponse.

Pseudoprogression can be defined as a subacute radiation-related side effect. It occurs after radiotherapy, particularly with high-dose delivery or with associated chemotherapy, occurring in the first 3 months after radiotherapy, or later, making identification and diagnosis difficult. This concerns about 20% of patients, with an incidence twice higher in patients with glioblastoma harboring a methylation of the *MGMT* promoter for which the prognosis is better. The pathophysiology is not well-understood, and some neurological symptoms may be associated. Spontaneous resolution is generally observed within a few weeks or months. No specific treatment is needed (19, 20), and these patients are therefore at risk of inappropriate further treatment. Radiation necrosis is a later and chronic inflammation radiation-related complication. This brain tissue injury occurs at least 3 months after completing radiotherapy, with a reported incidence from 5 to 40% (21). Clinical symptoms and imaging features may mime a relapse. Radiation necrosis lesions may be associated with recurrence lesions, making it difficult to diagnose conclusively (22). Biopsy is the gold standard but may not be feasible or may be inconclusive due to a limited and non-representative sampling. Furthermore, this invasive procedure may lead to further damage. Proposed treatments include steroids, bevacizumab, surgical resection, anticoagulation, or hyperbaric oxygen therapy.

Pseudoresponse is defined as an important diminution in contrast enhancement within the first two days after antiangiogenic therapies initiation. It is an indirect effect of treatment on vascular permeability but does not reflect a real antitumor effect (23, 24). The Macdonald criteria published in 1990 are based on the evaluation of tumor size measured on contrast enhancement (25). However, contrast enhancement is non-specific, reflecting only the extravasation of gadolinium through the disrupted blood–brain barrier (BBB). These response assessment in neuro-oncology (RANO) criteria added T2 and FLAIR modifications to contrast enhancement to evaluate tumor response (26). RANO has recently evolved into RANO modified and RANO in immunotherapy to take into account new treatments, such as targeted therapies and immunotherapy, and to allow standardized comparison in clinical trials (27, 28). Regardless of the evaluation criteria used, MRI techniques have some limitations, especially the confusion caused by treatment-induced modifications, such as radiation therapy, bevacizumab, or even corticosteroids that induce tumor shrinkage (29). More effective tools are needed to overcome these drawbacks and bring relevant additional information.

**Figure 1:**
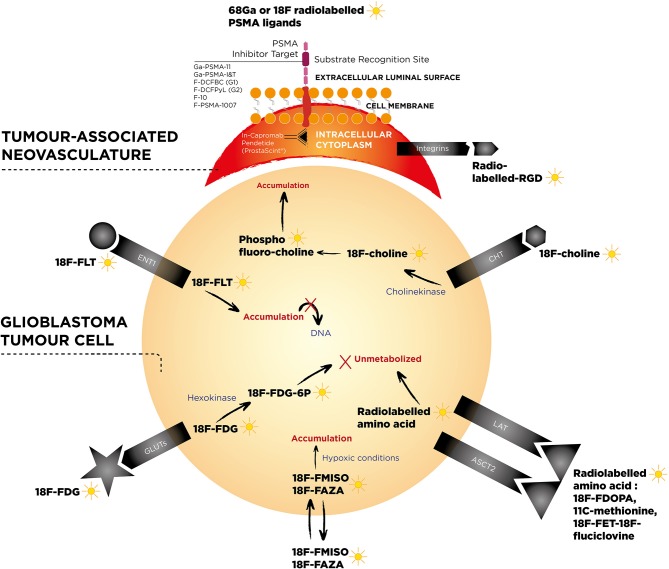
Pathophysiological mechanisms of main radiotracers used in glioblastomas investigation in functional nuclear imaging.

Functional nuclear imaging may investigate metabolism-related changes for oncological response assessment (30). Many positron emission tomography (PET) tracers have been studied, such as ^18^F-FDG (which explores glucose metabolism), the nucleoside analog ^18^F-fluorothymidine (^18^F-FLT), radiolabeled amino acids such as ^18^F-fluorodihydroxyphénylalanine (^18^F-FDOPA), ^18^F-fluoro-ethyl-tyrosine (^18^F-FET), ^11^C-methionine (^11^C-MET), and hypoxia tracers such as ^18^F-fluoromisonidazole (18F-FMISO) (Figure 1). These different tracers can provide additional reliable data in equivocal situations, guide a biopsy, help plan the tumoral volume delineation before radiotherapy, detect early tumor recurrence, distinguish pseudoprogression from relapse, evaluate response early during treatment (especially with new targeted treatments), and have prognostic value. Here, we detail the contributions that these tracers provide and we explore the opportunites that new tracers currently under evaluation may provide beyond routinely used tracers.

As explained above, the two major problems we are currently facing are the early distinction of tumor recurrence from post-therapeutic complications as well as the predictive response to targeted treatment. They imply both ethical and medico-economics issues, avoiding to inadequately discontinue an effective treatment or maintain an ineffective treatment.

## Carbohydrate Metabolism Tracer: ^18^F-FDG

The 2-deoxy-2-fluoro-D-glucose is a glucose analog used by tumors cells overexpressing GLUT1 and GLUT3 transporters in malignancies and is the most commonly used PET tracer in tumor assessment. After active transportation across the BBB, it is captured by the cells and phosphorylated as the first step of the Krebs cycle, preventing further release.

Despite its interesting role in differentiating low-grade from high-grade gliomas, no reliable cutoff level has been able to characterize brain tumors. The cutoff that Delbeke et al. proposed was not confirmed in semiquantitative analysis because of a large overlap between tumor standard uptake value (SUV)max and healthy parenchyma contralateral brain SUVmax (31–33). Furthermore, potential false positives, such as granulomatous diseases (tuberculosis or sarcoidosis), pyogenic abscesses, fungal infections, or other primary brain tumors, such as primary brain lymphomas, limited its role in initial and differential diagnosis (34–36).

It could however help in guiding the biopsy by highlighting the most hypermetabolic tumor area (37).

Its performances in discriminating radiation necrosis from tumor relapse are still debated (21). Assumptions for the use of ^18^F-FDG were based on an increase in metabolic rate in tumor recurrence, theoretically leading to a hot spot, compared to supposedly reduced metabolic rate in radiation necrosis leading to a cold spot (38). Initial studies reported strong performances (39–41), but the specificity varies greatly from one study to another (40–94%), while the sensitivity remains rather consistent (81–86%) (21). These differences could be explained by heterogeneity in the type of radiation, tumor heterogeneity or subtypes, and the timing of the PET examination after radiotherapy (29).

However, these encouraging performances have been revised downward. A study including the results of ^18^F-FDG PET vs. stereotactic biopsy demonstrated sensitivity and specificity of 43 and 100%, respectively, in the differentiation between recurrence and radiation necrosis (42). Indeed, PET interpretation based on the detection of an abnormal hypermetabolic or hypometabolic spot compared with the surrounding area or the contralateral equivalent tissue is challenging. The high physiological brain uptake limits brain tumor detection. False-positive lesions—inflammation, abscesses, foci of gliosis—and false-negative lesions, such as cerebral necrosis, limits reliability (43). Another issue is the frequent association of radiation necrosis with residual lesions (39). ^18^F-FDG is therefore disappointing in resolving this thorny issue (44). The utility of ^18^F-FDG would lie in the exploration of enhanced lesions on MRI to eliminate a differential diagnosis when clinical symptoms and MRI are discordant. A hypometabolic feature would allow the patient to be freed from biopsy or new treatments and would allow a simple follow-up (45).

Its prognostic and predictive roles are less controversial and better assessed. It is a recognized prognostic tool, regardless of its schedule in follow-up (46–48).

It is a significant independent prognostic factor for high-grade glioma before treatment (49) as well as an important predictive factor of survival in patients with suspicion of glioma relapse (50).

A ratio of 2.0 or 2.5 between the residual lesion SUVmax and the healthy white matter SUVmax could be used as a cutoff to identify patients with reduced survival who may potentially benefit from other therapeutic strategies (51).

Early changes in the glucose metabolic rate predicted response to temozolomide but failed to predict response to temozolomide plus radiotherapy (52). In treatment with bevacizumab and irinotecan, ^18^F-FDG is a predictive biomarker of response to treatment, with an uptake correlated with survival to a greater extent than any other prognostic factor tested (53).

**Figure 2:**
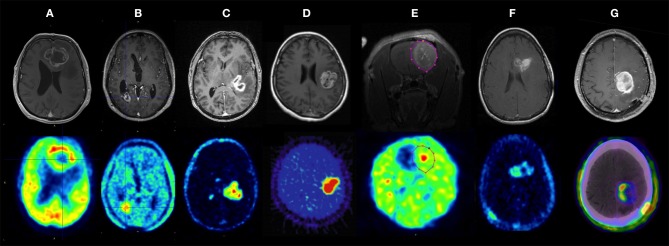
Contrast-enhanced MRI (top row) and multiple PET tracers (bottom row) in glioblastoma. **(A)** ^18^F-fluorodeoxyglucose (^18^F-FDG), **(B)** ^18^F-fluoroethyltyrosine (^18^F-FET), **(C)** ^18^F-fluoromethylcholine (^18^F-FCho), **(D)** ^18^F-fluoromisonidazole (^18^F-FMISO) PET in human glioblastoma, **(E)** ^18^F-fluoroazomycin arabinoside (^18^F-FAZA) PET of the rat F98 model, **(F)** ^18^F-fluorothymidine (^18^F-FLT) PET, and **(G)** ^18^F-AIF-NOTA-PRGD2 (18F-RGD) PET/CT in human GB. Bolcaen et al. (54). Used with permission from the publisher.

Its role in tumor delineation is limited by its lack of specificity, other tracers have a better performance in this indication (Figure 2) (54).

Dose escalation in radiation therapy, based on the ^18^F-FDG uptake, led to an additional boost in the hypermetabolic tumor areas. Although no additional toxicity was reported, this dose escalation did not improve PFS or OS in comparison to institutional historical series. Most recurrences occurred at sites that received the irradiation boost (55).

Several points have been stressed to improve PET with ^18^F-FDG in this disease. Delayed examinations could increase the examination performances by enhancing the tumor-to-normal brain uptake contrast. A better discrimination of tumors compared to the surrounding gray substance was reached on late images performed at 180–480 min both on visual and semiquantitative analysis. Imaging at 300 min (5 h) allowed an 18% increase in the tumor/gray matter ratio (T/G ratio). This could result from an improved degradation of ^18^F-FDG-6-phosphate in the normal cerebral parenchyma compared to the tumor. However, performing such delayed images in current clinical practice is rarely feasible (56).

It is of paramount importance to perform MRI to compare information. Hybrid PET/MRI could better allow to discriminate radiation necrosis from tumor recurrence by combining the PET parameters and MRI perfusion parameters (29, 57–59). The composite association of the apparent diffusion coefficient (ADC) on diffusion MRI with SUVmax/normal brain ratio on PET could also allow the identification of patients at risk of progression. Those with an ADC ≤ 1.400 × 10^−5^ mm^2^/s and with a SUVmax/Normal brain index >1.5 would be most at risk of tumor progression (60). Other authors reported that tumor cross-product and metabolic tumor volume (MTV) on PET at time of first recurrence were significantly correlated with survival (61).

## Cell Membrane Metabolism: ^18^F-Fluorocholine and ^11^C-Choline

Choline is a metabolite incorporated into cancer cell's membrane, which therefore reflects cellular membrane metabolism through its incorporation in different choline-transporting transmembrane systems, such as high-affinity choline transporters (CHTs), choline transporter-like proteins (CTLs), organic cation transporters (OCTs), and organic cation/carnitine transporters (OCTNs), and through the upregulation of choline kinase metabolizing choline to phosphatidylcholine. It can be radiolabeled with either ^18^F or ^11^C (62).

^11^C-choline is used to differentiate high-grade from low-grade gliomas but low-grade gliomas and non-neoplastic lesions failed to be distinguished (63). In single lesions enhanced on MRI, the uptake of ^18^F-fluorocholine was significantly higher in high-grade gliomas compared with benign lesions with SUVmax lesions (1.89 ± 0.78) vs. (0.59 ± 0.31), respectively (*p* < 0.0001). The increased uptake in the peritumoral area was characterized high-grade gliomas (64). However, investigations on malignant and non-malignant lesions from brain and other locations showed that uptake may occur in lesions other than malignant ones, and recommended caution in interpreting results (65). For example, benign lesions, such as tumefactive demyelination and radiation-induced mass, may be the site of moderate to significant ^18^F-fluorocholine uptake (66).

^18^F-Fluorocholine may be useful in differentiating tumor recurrence from radiation necrosis. There is a very low uptake in acute radiation necrosis in rats based on disruption of the BBB and uptake by inflammatory cells, especially macrophages. In humans, radiation necrosis differs because dose and type of irradiation are obviously different, and the onset of a chronic phase of radiation injury is frequently observed, but the use of ^18^F-fluorocholine is nevertheless considered (67) and reinforced with results obtained from 55 patients with suspected brain tumor recurrence or radiation necrosis after radiation therapy, including initial grade-IV gliomas. ^11^C-choline exceeded the performance of ^18^F-FDG, with sensitivity and specificity of 92.3 and 76.9%, and 87.5% and 62.5%, respectively. However, histologic confirmation was performed in only a few patients. In addition, two false positives—gliosis and post-radiation granuloma—and three false negatives were reported, including two negative glioblastomas attributed to a too short delay after the end of radiotherapy (68).

## Radiolabeled Amino Acid

Amino acid incorporation into membrane transporters is upregulated in tumor cells, regardless of the permeability of the BBB. Their contribution can outperform the conventional and ^18^F-FDG limitations and drawbacks, in particular for contentious cases (69). The intense uptake in tumors and weak capture in the normal brain provide a higher contrast and better delineation between tumors and healthy surrounding tissue (29). The non-specific increase in amino acid results from enhanced energy requirements, increased cell division and protein synthesis, associated with specific oncogenic modifications in the targeted membrane transporters (70). An increased expression of receptors in the vasculature of tumor lesions also contributed to amino acid uptake (71). The use of radiolabeled amino acids, mainly ^11^C-methionine, ^18^F-FDOPA, and ^18^F-FET in gliomas, and more specifically glioblastomas, is actively being investigated. The RANO working group and the European Association for Neuro-Oncology published recommendations for the use of these PET tracers in routine clinical practice to differentiate neoplastic and non-neoplastic lesions, define tumor extent before surgical resection, evaluate the quality of resection with post-operative control and search for tumor residue, determinate biopsy site, assess prognosis before or after treatment, determine MTV as part of radiotherapy planning, perform therapeutic evaluation of adjuvant treatments, differentiate post-therapeutic modifications and tumor recurrence, and monitor adjuvant therapy (69–72).

## ^11^C-Methionine

The first radiolabeled amino acid explored was ^11^C-methionine, however, the short half-life of 20 min for ^11^C restricts its use to the rare facilities with on-site cyclotron.

Contrary to ^18^F-FDG, which does not allow benign lesions to be differentiated from malignant lesions in the case of iso- or hypo-metabolic lesions, performances for ^11^C-methionine are excellent (sensitivity and specificity of 89 and 100%, respectively). Moreover, ^11^C-methionine binding correlates with the proliferation index (73).

^11^C-methionine allows the distinction between low-grade and high-grade tumors. Lopci et al. showed a more intense ^11^C-methionine uptake in primary glioblastoma compared to other gliomas. The captation significantly correlated with *IDH1* mutation status in the majority of gliomas, with the exception of glioblastomas (74).

It may a good substitute for ^18^F-FDG in guiding stereotactic biopsy owing to better performances in sensitivity and specificity, especially in lesions with no ^18^F-FDG uptake or uptake less than or equal to that of gray matter (75, 76).

Conflicting results remain in differentiating between radiation necrosis and tumor recurrence. ^11^C-methionine was first identified as better than ^18^F-FDG and ^11^C-choline (77). However, Kim et al. failed to prove a significant difference in discriminating radiation necrosis from tumor recurrence. The uptake in a radiation necrosis lesion may result from an increased methionine metabolism and permeability induced by reactive gliosis after radiation injury, an accumulation in glial cells proliferating in the area of radiation necrosis and BBB modifications (78).

It allows the refining of irradiation and reirradiation planning. It may help identify areas at high risk of recurrence that may benefit from a radiation boost. The fusion of PET with ^11^C-methionine, computed tomography (CT), and MRI results could be a potential tool in the reirradiation of glioblastoma recurrences. The target volume delineation may be improved by better discriminating post-therapeutic modifications of tumor zones to spare healthy tissues and thus improve the therapeutic ratio (79, 80). This fusion-based radiation planning significantly improved the survival in reirradiated patients compared with patients for whom radiation planning was based on CT/MRI alone (81).

It contributes to the management of patients undergoing treatment with temozolomide. A small series reported that tumor response could be evaluated after three cycles of chemotherapy, with the absence of progression correlated with tracer uptake stability during the next three cycles. The stable or decreased uptake on metabolic imaging was consistent with clinical stability. A decreased uptake was associated with a significantly longer time to progression (TTP) compared with increased uptake (82). ^11^C-MET-PET at 8 weeks allowed identification of responders with significantly longer PFS than non-responders (83).

Wider use of ^11^C-methionine is nonetheless limited by the need for an on-site cyclotron. Other radiolabeled amino acids have therefore been explored to overcome this restriction.

## ^18^F-FDOPA

^18^F-FDOPA was initially used to assess the distribution of dopamine in patients with movement disease as a precursor to dopamine entering into brain tumors through the L-type amino acid transporter (LAT), without significant uptake in the surrounding brain parenchyma, except the basal ganglia (84). The potential interest in ^18^F-FDOPA in the exploration of brain tumors was first reported by accidentally discovering a grade II oligo-astrocytoma with ^18^F-FDOPA in a patient with movement disorders and suspicion of underlying Parkinson's disease (85).

^18^F-FDOPA uptake is significantly associated with LAT-1 expression, but the linear correlation between the LAT-1 expression level and the intensity of fixation is still debated (86, 87). ^18^F-FDOPA transported into tumor cells does not remain trapped. The permeability of the BBB could have an additional and complementary effect and may be added to the increased expression of amino acid carriers in high-grade tumors (88).

^18^F-FDOPA combines the successful biopharmacological properties of amino acids and the convenient physical and logistical properties of fluorine 18. It could replace ^11^C-methionine in amino acid transport imaging in glioblastomas. Visual analyses and SUV ratios of ^18^F-FDOPA compared with ^11^C-methionine suggest that it is an excellent surrogate for the exploration of recurrent lesions, especially for centers without on-site cyclotron. ^18^F-FDOPA may assist in grading newly diagnosed gliomas, in planning radiotherapy, and in assessing treatment response (89). Another advantage is the possibility of acquiring images as early as 20 min after injection, without hindrance from the delayed basal ganglia physiological fixation, which peaks later (85, 90, 91).

18F-FDOPA is used to discriminate high grades from low grades. 18F-FDOPA uptake prior to the treatment of newly diagnosed glioma has been reported to correlate with tumor grade and proliferation. No significant correlation was reported in recurrent gliomas (92). The correlation between uptake and tumoral aggressiveness can help in the distinction of low- and high-grade tumors, according to enhancement. Distinct, although close, SUVmax thresholds have been reported (92, 93). It should be noted that glioblastoma tumor uptake may sometimes be less intense than oligodendroglioma uptake (94). Janvier and collaborators highlighted that other indices, such as the SUVmean tumor/normal brain ratio or the SUVmean tumor/striatum ratio could allow a better discrimination between low- and high-grade tumors in routine practice (93). ^18^F-FDOPA kinetics and capture revealed differences between high- and low-grade tumors, notably differences in time–activity curves. High-grade tumor profiles showed an early maximum followed by a steep decrease, whereas low-grade tumors showed a slowly declining curve (88). Conversely, some rare studies failed to demonstrate a difference in ^18^F-FDOPA uptake between low-grade and high-grade gliomas (95, 96), possibly related to limited statistical power and differences in examination time and duration for image acquisition.

18F-FDOPA to guide the biopsy site: the intensity of uptake is related to the grade. Hence, 18F-FDOPA could identify high-grade areas and could determine sites that could benefit from radiotherapy boost (97).

However, 18F-FDOPA in planning radiotherapy was disappointing. It allowed broader tumor delineation and significantly wider contour of the gross tumor volume (GTV) than that defined with MRI alone; however, the therapeutic impact was low as almost all relapses occurred outside the PET-GTV (98, 99).

^18^F-FDOPA is very useful for distinguishing radiation necrosis and glioblastoma recurrence (100, 101). Visual analysis based on a 5-point visual scale or semiquantitative images analysis using lesion-to-striatum or lesion-to-normal brain tissue were accurate in distinguishing recurrence from treatment-related changes in 110 patients followed for glioblastoma and were prognostic of PFS. Patients with positive examinations had a 4.2 times shorter median OS than patients with negative examinations (101).

**Figure 3:**
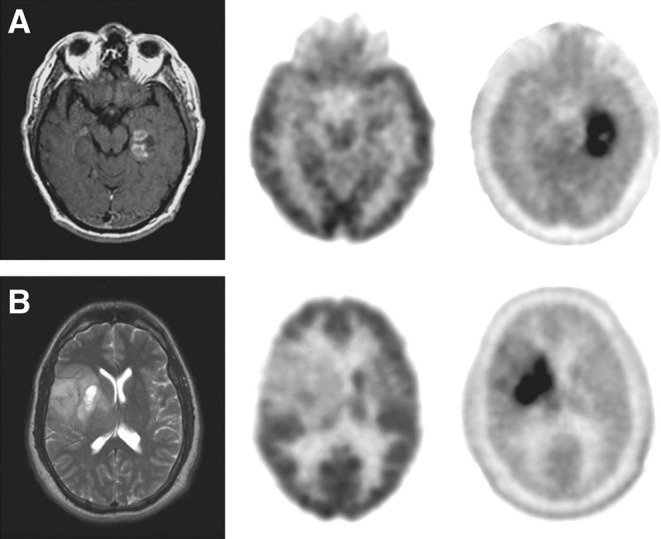
MRI (left), ^18^F-FDG PET (middle), and ^18^F-FDOPA PET (right) of newly diagnosed tumors. **(A)** Glioblastoma. **(B)** Grade II oligodendroglioma. This research was originally published in Chen et al. (95).

The superiority of ^18^F-FDOPA over ^18^F-FDG is reported in the recurrent tumor evaluation and in the differentiation between tumor recurrence and radiation necrosis, thanks to a higher contrast between tumor tissue and normal tissue. Sensitivity of ^18^F-FDOPA was higher than ^18^F-FDG on visual analysis, but comparable, and mediocre specificities were reported. The addition of a semiquantitative analysis through a ratio determination for ^18^F-FDOPA [tumor/striatum ratio (T/S), tumor/normal white matter ratio (T/W), and tumor/contralateral normal brain tissue ratio (T/N)] improved specificity. A T/S ratio of 0.75 resulted in a maximum sensitivity of 100% with a specificity of 86%, while a T/S ratio of 1.0 resulted in a small decrease in sensitivity (92%) with a good specificity of 95%. A 1.0 threshold in a first-line assessment or clinical suspicion of radiation necrosis was predominant, and the 0.75 threshold in inconclusive cases or with the suspicion of tumor recurrence as the most likely hypothesis (Figure 3) (95). Another study confirmed these results, with better specificities (100).

Rare false-negative glioblastoma and a few false-positive lesions have been reported, such as acute disseminated encephalomyelitis, neurosarcoidosis, demyelinating lesions, and inflammatory granulations on the resection margins, through activation of macrophages after surgery. Therefore, a weak homogeneous circumferential fixation on the resection margins should be considered with caution, as it may be caused by post-operative inflammatory changes rather than in association with tumor recurrence and may be closely monitored (91, 102, 103).

^18^F-FDOPA as a prognostic factor: The tumor to normal tissue (T/N) ratio significantly correlated with survival in patients suspected of glioma recurrence (104). In low-grade gliomas, intensity of uptake may be an independent predictive factor of disease progression and its prognostic role was proposed (105, 106). However, unexpected and somewhat paradoxical results showed a higher capture of ^18^F-FDOPA by IDH*-*mutated grade II and III gliomas compared to wildtype ones (107). Similarly, a high ^18^F-FDOPA uptake was predictive of a low growth rate tumor, regardless of IDH status (108).

^18^F-FDOPA is also used to evaluate tumor response in patients with recurrent high-grade glioma treated with anti-angiogenic therapy, such as bevacizumab. The absolute MTV measured 2 and 6 weeks after the treatment initiation correlated with the tumor response (109). This predictive value based on parametric response maps showed a correlation between evolution and both PFS and OS (110).

As with ^18^F-FDG, results should be interpreted in conjunction with MRI results and ideally using merged images if possible. In rare cases, ^18^F-FDOPA may detect recurrences earlier than MRI. Inverse association between the ^18^F-FDOPA uptake level and ADC and a proportional association between the ^18^F-FDOPA uptake level and the mitotic index have been reported (102, 111).

The ^18^F-FDOPA PET can therefore modify the management of patients with glioblastoma owing to its performances in diagnostic, therapeutic, and prognostic evaluation (89). The main drawbacks remain its cost and its availability (112).

## ^18^F-FET

To overcome the limitations for the use of ^11^C-methionine, ^18^F-labeled amino acids have been synthesized, such as ^18^F-FET.^18^F-FET enters tumor cells through a specific amino acid transport system (LAT) and is not metabolized or incorporated into proteins.

^18^F-FET and ^11^C-methionine showed equivalent performances in lesion detection to differentiate post-therapeutic modifications from recurrences and to confirm delineation of MTV (113). Compared with ^18^F-FDOPA, no significant difference was reported in visual analysis for patients either with primary or recurrent high-grade glioma. Semiquantitative analysis outlined a significant difference; however, it had no impact on the delineation of tumor volume (114).

^18^F-FET clinical applications included guiding biopsy, tumor delineation, scheduling and monitoring treatment (surgery or radiotherapy), and distinguishing between radiation necrosis and tumor recurrence.

^18^F-FET to target the biopsy area in patients with characteristic glioma lesions on the MRI. A lesion was considered to be suspicious of tumor when it displayed a brain/ratio >1.6 with ^18^F-FET. The addition of PET ^18^F-FET and MR spectroscopy to conventional MRI improved the efficiency of biopsy targeting (115).

^18^F-FET results used in conjunction with MRI in tumor delineation. Tumor delineation showed larger volumes with ^18^F-FET than relative cerebral blood volume (rCBV) on perfusion-weighted MRI (116). Combined with MRI, it would better define the areas where tumors are likely to recur after radiotherapy treatment (117). It also identifies post-operative residual tumors in a more sensitive way than MRI (118). Investigating this setting through a dual time-point imaging of FET uptake at 10 and 60 min after radionuclide injection, some authors showed that it was less time-consuming than most dynamic data acquisition protocols but still overcomes many of the limitations of static acquisition (119).

Differentiation between low- and high-grade gliomas. One study reported no significant discrepancy between these tumors, while another retrospective study reported that a tumor-to-brain max ratio <2.5 could rule out a high-grade tumor with a high probability (120). Some authors have highlighted the potential contribution of dynamic acquisition. In a study based on the SUVmax lesion/background ratio, low-grade and high-grade gliomas showed statistically different uptakes and could be distinguished with a 2.58 threshold. Notwithstanding, there was a significant overlap limiting the relevance of these tools. Conversely, the dynamic acquisitions allowed a better discrimination through the time–activity curve, with an early peak at 10–20 min for high-grade gliomas, followed by a decrease, vs. a sluggish growth for low-grade gliomas (121, 122). Nevertheless, this dynamic acquisition takes two to three more times than a static acquisition, making it difficult to implement as routine. Early static acquisition performance at 5–15 min is sufficiently consistent to distinguish low- and high-grade tumors. The tumor-to-brain ratio (TBR) was more accurate on such early images than at 20–40 min, thanks to a higher ratio for high-grade tumors on the early images. However, the calculation of this early ratio remained less precise than the dynamic acquisition for this objective (123). Although it is a powerful tool to distinguish low- and high-grade glioma, histological analysis remains the gold standard (124).

^18^F-FET for the differentiation between post-therapeutic modifications and relapses. ^18^F-FET is useful to distinguish recurrence from radiation necrosis, with its low uptake after acute radiation necrosis due to BBB leakage. The ratio between the radiation necrosis captation and the normal cortex uptake is lower for ^18^F-FET compared with ^18^F-FDG and ^18^F-fluorocholine (67).

Metabolic characteristics were explored in patients with gliomas after treatment with various therapeutic approaches and suspicion of recurrence. An intense and focal uptake suggests relapse, whereas a weak homogeneous uptake on the resection bed was rather in favor of benign post-therapeutic modifications (125).

However, these results are still being debated, and Mehrkens and collaborators adjusted these results. The examination could not replace the stereotactic biopsy (126).

The additional dynamic data mentioned above can help in differentiating low-grade from high-grade glioma tumors but also allows the discrimination of recurrences from radiation necrosis and would have a prognostic contribution. A mean tumor/brain ratio ≥2.0 on static images or a time-to-peak <45 min on dynamic images improves the accuracy in the discrimination of glioma recurrences from post-therapeutic changes compared to MRI. It would outperform MRI in this setting (127–129).

^18^F-FET can also be used to differentiate an early progression after a radiochemotherapy completion from a pseudoprogression, with an uptake significantly higher in early progression than in pseudoprogression. The optimal TBR maximal threshold value for pseudoprogression was 2.3. The patients with pseudoprogression had significantly longer OS than the patients with early progression (130). The dynamic acquisition would also identify a late pseudo progression, defined as occurring beyond 3 months after completion of radiotherapy (131).

Despite its performances in differential diagnosis between the post-therapeutic modifications and recurrent changes, its specificity and image analysis remain somewhat controversial and debated. Increased amino acid tracer uptake may also occur in non-neoplastic lesions or processes (e.g., ischemic stroke, local infections related to a brain abscess, inflammatory processes such as multiple sclerosis, status epilepticus) (132–135).

Prognostic role of ^18^F-FET: The MTV was identified as an independent prognostic factor. A small MTV before any treatment or just after surgery before radiochemotherapy correlated with an increased OS and PFS (136–138). A TBR threshold ratio of 1.6 would correlate with OS and with disease-free survival (DFS), and would allow—after surgery and before adjuvant radiochemotherapy—the residual tumor volume to be accurately determined in order to improve the delineation. Similarly, an increased time–activity curve at baseline examination is a factor associated with a prolonged OS (137). ^18^F-FET has a predictive role in early response to radiochemotherapy. The patients with an ^18^F-FET uptake decrease >10% estimated by the TBRmax on an examination performed 7–10 days after treatment discontinuation would have a longer median OS (139).

These results were relatively consistent with those of other studies (140). Nevertheless, the modification of the kinetics parameters on the dynamic acquisition during treatment had no prognostic impact (141).

^18^F-FET complementary role to MRI for therapeutic response assessment: Response to bevacizumab and irinotecan therapy in patients with high-grade glioma recurrence was discordant in 40% of cases. ^18^F-FET showed tumor progression earlier than MRI. A PET response—defined as a reduction of more than 45% of the metabolically active tumor volume, that is, with a TBR ≥1.6—was observed at an early stage. This PET response significantly correlated with increased median PFS and OS. A TBR reduction ≥17% at follow-up allowed the discrimination of responders (PFS ≥6 months) from non-responders (PFS ≤ 6 months) with good performances. Interestingly, at baseline, kinetics characteristics, such as an early peak of capture followed by a decrease, were more often observed in non-responder patients (142). A few years earlier, a similar study, using the same response criteria, had found consistent results. In more than one third of the cases where MRI failed to demonstrate progression according to the RANO criteria, ^18^F-FET PET detected progression. Survival was significantly higher in responders than in non-responders (143). This approach could be cost-effective while avoiding continuation of ineffective treatment with associated side effects and costs. These medico-economic aspects should also be considered in the area of personalized medicine, where costs associated with new treatments, such as targeted treatments and immunotherapy, are soaring (144).

^18^F-FET may contribute to improving management of patients pursuing a tumor-treating field (TTF) therapy. This innovative treatment consists of using transducers to deliver an electric wave, transformed into electromagnetic energy to the scalp. The low-intensity and intermediate-frequency waves (200 kHz) generated in the brain are toxic to cells. It prevents neoplastic cell division and causes neoplastic cell death, with no significant effect on normal quiescent cells. This adjuvant therapy with temozolomide improves PFS and OS in patients who have completed initial radiochemotherapy treatment. The ^18^F-FET TBRmax and TBRmean ratios have been proposed to discriminate relapses from post-therapeutic changes (145).

The recent advances are no longer limited to studying the standard static and dynamic parameters of ^18^F-FET. Radiomics parameters based on textural features can be associated with reflect tumor heterogeneity, with good performances in the subgrading of high-grade gliomas (grade III vs. grade IV); they have a predictive role for tumor progression and correlate with OS (146). Kebir and collaborators highlighted their role in the detection of pseudoprogression (147). In addition, the combination of conventional static and dynamic criteria with radiomic parameters allows a non-invasive prediction of IDH status with good performances (148). The current drawbacks of such parameters are that textural feature analysis is influenced by image quality, especially by the spatial resolution, and post-processing of image data is complex and time-consuming.

According to Galldiks and collaborators, ^18^F-FET should be preferred to other radiolabeled amino acids because of its logistical advantage, thanks to its ^18^F radiolabel, the absence of striatum uptake, and the accurate discrimination between high- and low-grade glioma with kinetics parameters from dynamic acquisition (149). However, it is not available in all countries.

## Radiolabeled Nucleoside Analog: ^18^F-FLT

^18^F-FLT is a thymidine analog that is phosphorylated by thymidine kinase-1, an enzyme whose activity is increased in tumor cells, and that operates during the DNA synthesis. However, this substrate is not incorporated into DNA (112). ^18^F-FLT capture requires the rupture of the BBB and reflects cell proliferation—more precisely, the fraction of tumor cells in the S phase (150).

The use of ^18^F-FLT in grading gliomas: Glioblastomas display a higher uptake ratio than astrocytomas (*p* < 0.01) (151). ^18^F-FLT uptake significantly differs according to the lesion/background ratio in high- and low-grade tumors from different brain benign and neoplastic lesions. The proliferation index (Ki-67) correlates with the ^18^F-FLT fixation in gliomas. However, ^18^F-FLT failed to distinguish non-tumor lesions from low-grade tumors (152). ^18^F-FLT use was more efficient than ^18^F-FDG in detecting recurrences of high-grade tumors, predicting tumor progression and survival in the follow-up of patients with low- and high-grade gliomas, and ^18^F-FLT results correlated with the Ki-67 proliferation index (153).

Role of ^18^F-FLT in discriminating radiation necrosis from tumor recurrence: Enslow and collaborators have demonstrated the potential use of ^18^F-FLT in patients with suspected recurrence of treated gliomas of grade ≥II: visual and quantitative analysis allows distinction between radiation necrosis from tumor recurrence. However, ^18^F-FLT failed to prove its superiority in this setting compared to ^18^F-FDG (154).

The prognostic role of ^18^F-FLT. The proliferative volume is a prognostic factor before treatment. The proliferative volume assessed through the signal-to-background ratio (SBR) for an adaptive threshold delineation (PVSBR) segmentation method in patients followed up for high-grade gliomas was significantly associated with OS compared with SUVmax or with the proliferative volume estimated according to other segmentation methods (155).

The predictive role of ^18^F-FLT in assessing response to treatment: Metabolic changes at 2 and 6 weeks after treatment initiation significantly correlated with PFS and OS in patients followed for glioma after bevacizumab administration, the metabolic response being defined as a decrease equal to or greater than 25% in tumor ^18^F-FLT standardized uptake values from baseline. The subsequent median OS was 3.3 times longer in responders than in non-responders. ^18^F-FLT PET was more predictive than MRI for early response to treatment (156). These results confirm prior results in recurrent malignant gliomas treated with bevacizumab and irinotecan. Whether earlier imaging at 1–2 weeks could be a predictive factor as accurate as later imaging at 6 weeks remains to be confirmed (157). Dynamic acquisitions have shown their interests in predicting survival in patients followed for recurrent glioma treated with bevacizumab and irinotecan. The prognostic value of kinetic parameters of ^18^F-FLT, estimated 2 weeks after the beginning of the treatment, was more relevant than that of ^18^F-FDOPA (158). The parametric response maps modifications correlated with PFS in patients treated with bevacizumab. However, the ^18^F-FLT was not associated with OS (110).

## Hypoxia Imaging: ^18^F-FMISO and ^18^F-FAZA

^18^F-FMISO is a hypoxia tracer. It is a nitro-imidazole derivative whose metabolites are blocked in hypoxic cells. It is a positive tracer of hypoxia; its uptake is inversely proportional to the partial pressure in oxygen. It diffuses into the cells, is reduced in the case of hypoxia, and then remains trapped in the cells. Its role could be crucial because hypoxia is related to tumor aggressiveness and resistance to radiation treatment. Its uptake does not depend on the strength of the tumor perfusion or on the BBB permeability (159). Its uptake is significantly correlated with the Ki-67 proliferation index (160).

Its main drawback is its lipophilicity, which generates a high background activity and requires both early and delayed acquisitions (112).

^18^F-FMiso is complementary to MRI. ^18^F-FMISO uptake straddles the peripheral area of the anomalies found in T1 gadolinium, supporting the principle that local hypoxia leads to the production of angiogenic factors, themselves leading to the creation of neovasculature (161).

Use of ^18^F-FMISO to discriminate glioblastomas from other gliomas: An intense and significant fixation relative to surrounding cerebral background has been reported in patients with glioblastomas compared with those with other gliomas. The sensitivity and specificity in glioblastoma detection were higher using ^18^F-FMISO than using ^18^F-FDG. Similarly, the lesion/cerebellum ratio was higher in patients followed for glioblastoma than in non-glioblastoma patients, without overlap between these two groups (162).

Hypoxia measured on ^18^F-FMISO PET as prognostic factor: An increase in SUVpeak before treatment with radiochemotherapy was associated with a shorter OS in patients followed for glioblastoma (163). Similarly, the intensity and volume of tumor hypoxia in glioblastomas are significantly correlated with time to progression and survival (162, 164, 165).

^18^F- Fluoroazomycinarabinofuranoside (^18^F-FAZA), another tracer of hypoxia, displays a better signal-to-noise ratio due to less lipophilic properties and greater clearance. It may help identify small hypoxic tumor areas confined in hypometabolic necrotic areas, as revealed with ^18^F-FDG. These data would be of potential interest for radiotherapy planning (166).

Hypoxia is a poor prognostic factor, causing resistance to radiation therapy, leading to poorer local control. Using these hypoxia tracers, identification of these intratumor hypoxic areas would make it possible to increase radiation doses to the whole tumor or to specifically target the hypoxic zones (167). However, it should be noted that there is temporal and spatial variability of small hypoxic and necrotic zones, which could compound the role and contribution of hypoxia imaging in tumor exploration (168).

## New Tracers Under Development in This Setting

Each aforementioned tracer has shortcomings (Table 1), which hamper PET imaging in playing a major role in glioblastoma management (169). The unexpected role of the transmembrane glycoprotein prostate-specific membrane antigen (PMSA) encoded by the *FOHL1* gene, and overexpressed in prostate adenocarcinomas, may overcome some drawbacks. Radiolabeling of PMSA with the positron emitter gallium 68 is readily available, thanks to a 68Ge/68Ga on-site generator. It can also be radiolabeled with ^18^F, such as ^18^F-DCFPyL or ^18^F-PSMA-1007, but synthesis with cyclotron sites is not achievable (170). PMSA can be targeted with small molecular ligands, such as 68Ga-(HBED-CC), also named 68Ga-PSMA-11. The key role of this PMSA in prostate cancer care tends to replace ^18^F-fluorocholine PET/CT and bone scan to become a standard (171–175).

**Table 1:** Advantages and drawbacks of main radiotracers used in gliomas investigation in functional nuclear imaging.

	**Physiopathology**	**Advantages**	**Drawbacks**
18F-FDG	Carbohydrate metabolism	- Availability - Help in guiding biopsy - Prognostic role - Predictive role	- Physiological brain fixation (false negative) - Lack of specificity (false positive) - No differentiation between low- and high-grade lesion - Disappointing in recurrence from radiation necrosis differentiating - Disappointing in radiotherapy planning
11C-choline and 18F-Fcholine	Cell membrane metabolism	- Grading gliomas - - Recurrence from radiation necrosis differentiation	- Rare false positive and false negative - No differentiation between low-grade gliomas and non-neoplastic lesions
11C-methionine	Amino acid transport	- Grading gliomas - Help in guiding stereotactic biopsy - Radiotherapy planning - - Predictive role	- Limited use to on-site cyclotron - Controversial and results in differentiation between radiation necrosis and tumor recurrence
18F-FDOPA	Amino acid transport	- Logistical advantage thanks to its ^18^F radiolabel - Early acquisition - Grading gliomas - Guiding biopsy - Radiotherapy planning - Recurrence from radiation necrosis differentiation - Prognostic role - Predictive role	- Disappointing in radiotherapy planning - Availability compared to radiolabeled -PSMA
18F-FET	Amino acid transport	- Logistical advantage thanks to its ^18^F radiolabel - No striatum uptake - Dynamic acquisition - Grading gliomas - Guiding biopsy - Radiotherapy planning - Recurrence from radiation necrosis differentiation - Prognostic role - - Predictive role	- Availability
18F-FLT	DNA synthesis	- Grading gliomas - Prognostic role - Predictive role	- No distinction between non-tumor lesions and low-grade tumors - Compared to 18F-FDG no superiority in discriminating radiation necrosis from tumor recurrence
18F-FMISO and 18F-FAZA	Hypoxia imaging	- Discriminating glioblastomas from other gliomas - Prognostic role - Radiotherapy planning: boost on small hypoxic tumor areas	−18F-FMISO: requires realization of early and delayed acquisition due to a high background activity
68Ga-PSMA and 18F-PSMA	Endothelium of tumor-associated neovasculature imaging	- Differenciation between radiation necrosis and recurrence - Identification of residual disease immediately after surgery - Possible role in characterizing high and low-grade glioma - Role in peptide therapy to be investigated	- Cerebral necrosis as a possible pitfall and possible false positive

PSMA is not specific to prostate cancer. PSMA is highly expressed in many other cells, such as renal proximal tubular epithelium and duodenum columnar epithelium. PMSA is also expressed in the endothelium of tumor-associated neovasculature but never in malignant tumor cells themselves nor in normal vessels (176–178).

This was confirmed in a recent study, which demontrates that two thirds of glioblastoma neovasculatures express PSMA with a high and moderate intensity in most cases, with no correlation between PSMA expression and endothelial proliferation (179).

Glioblastoma multiformes, among the most vascularized tumors, also express PSMA, which could represent a new target of choice. Compared to glioblastomas, grade II and III gliomas have a weaker expression, with predominant expression located not on vessels, but on astrocytes. Normal cerebral parenchyma expresses little or no PSMA either in brain cells or vessels (180, 181). The PSMA may be implicated in angiogenesis through its participation in the destruction of the extracellular matrix during the invasion by neovessels and modulation of the integrin transduction signal in a complex way (182). Its folate activity may facilitate angiogenesis and vasculogenesis by increasing local folic acid availability.

However, Gordon and collaborators reported a PSMA overexpression in some non-neoplastic neovasculatures involved in repair and regeneration mechanisms, such as granulation tissues and scars (183).

**Figure 4:**
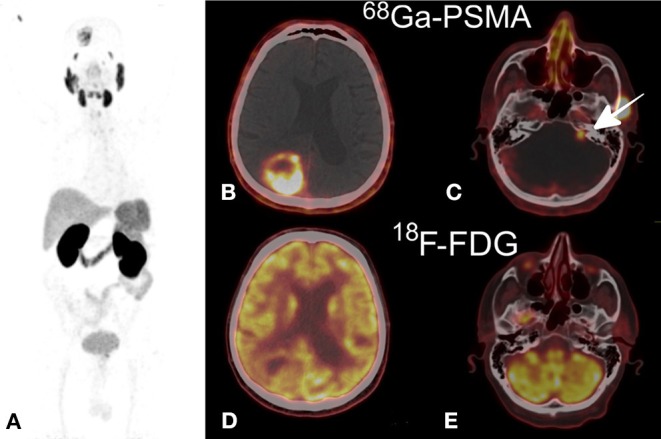
**(A)** Maximum intensity projection ^68^Ga-PSMA (MIP), **(B,C)** ^68^Ga-PSMA axial PET/CT fusion demonstrating a non-homogeneous uptake in the right parietal mass **(B)** and a lower uptake in the left auditory neuroma **(C)**, **(D,E)** ^18^F-FDG axial PET/CT fusion showing an increased uptake comparable to that in the gray matter in the parietal tumor **(D)** and no uptake in the neuroma **(E)**. This research was originally published in Kunikowska et al. (186).

The visualization of glioblastoma with ^68^Ga-PSMA has previously been reported and is a potential false positive and can be the source of pitfalls (184, 185). A few case reports have shown glioblastomas with PSMA radiolabeled with either 68Ga or 18F, the intense uptake being consistent with contrast-enhancing tumor on MRI (Figure 4) (186–188).

Sasikumar and collaborators compared ^68^Ga-PSMA PET/CT and ^18^F-FDG PET/CT in a series of 10 patients followed for various brain tumors, including five patients suspected of glioblastoma recurrence, with the aim to characterize the lesions or to make the differential diagnosis between radiation necrosis and recurrence. The authors highlighted the superiority of ^68^Ga-PSMA over ^18^F-FDG, owing to an advantageous target-to-background ratio. The four glioblastoma relapses were correctly and easily identified (189).

The same team later published a series of 15 patients followed for gliomas, including 10 glioblastomas, imaged with ^68^Ga-PSMA PET/CT. The authors confirmed previous results in patients for post-treatment recurrence assessment. The examination also identified the residual disease immediately after surgery more clearly than MRI.

However, a case of cerebral radionecrosis demonstrating ^18^F-DCFPyL uptake has been published, highlighting this entity as a possible pitfall while interpreting this examination (190).

Characterizing high- and low-grade glioma in treatment-naive patients, Verma et al. demonstrated higher ^68^Ga-PSMA-11 uptake and tumor-to-background ratio in high-grade glioma than in low-grade ones. PSMA SUVmax and MIB-1 both correlated with the tumor grade (191).

Further evidence from robust studies are needed to define the precise role of this imaging in the evaluation of gliomas (evaluation of recurrences, radiotherapy planning, and immediate post-surgery restaging, among others) (192).

Whether it could be used in peptide therapy with α or β- emitters is still being discussed. The effectiveness of such a treatment could be reduced by the distance to be covered by the β- to reach the tumor cells, the PSMA being expressed by the neovessels, and not by the glial cells themselves. The absence of tracer internalization in glial cells hinders its theranostic use. The radiation on the neovessels and its potential tumoricidal efficiency can only be speculated. Dosimetric studies are required (186, 193).

Many other tracers have been proposed and studied (57).

Matsuda and collaborators successfully imaged PSMA expression in three patients followed for recurrent high-grade gliomas and brain metastases using ^89^Zr-Df-IAB2M anti-PSMA minibody and outlined a trend for the binding intensity of this tracer to correlate with PSMA expression level in tumor vessels. This tracer may play a role in distinguishing between post-therapeutic recurrence and radiation necrosis, as well as in predicting efficacy and evaluating tumor response under bevacizumab (194).

Somatostatin receptors, especially SSTR2 subtype, are overexpressed in neuroendocrine tumors, allowing their visualization with ^68^Ga-radiolabeled somatostatin analogs, such as ^68^Ga-DOTATATE. These receptors are also overexpressed in activated macrophages, leading to false-positive inflammatory lesions. Some authors studied the potential use of ^68^Ga-DOTATATE to assess glioblastomas that belong to the wide group of tumor-associated macrophages. They failed to prove the interest of ^68^Ga-DOTATATE because of a weak SSTR2A expression on the cell surface of infiltrating macrophages and on tumor cells (195).

Other investigations explored the interest of radioguided surgery with β- isotopes in high-grade glioma. The aim of this technique, based on the same rules as sentinel node mapping, is to help the surgeon during resection to evaluate the extent of the resection and to maximize the resection margins while minimizing the peripheral parenchyma resection surrounding the lesion. A tumor-specific tracer, in this case ^90^Y-DOTATOC, was injected before surgery. Its radiolabeling with a β- emitting isotope makes it possible to get rid of the background noise obtained with a gamma isotope. Specific detection probes were used to collect information in real time. Although it was less interesting than in meningioma because of a lower uptake, the tumor-to-non-tumor ratio was strong enough to support the use of this technique in high-grade gliomas (196). The place of this tracer in glioblastoma evaluation remains uncertain, with probably less potential impact than for meningiomas, due to the low expression of these receptors by tumor cells.

^11^C-alpha-methyltryptophan (AMT) could be an alternative to ^11^C-methionine, but this does not avoid the need for on-site cyclotron (57). It has demonstrated its value in both discrimination between radiation necrosis and recurrence and in the detection of post-therapeutic recurrences. This tracer is accumulated in cells of gliomas and metabolized by the kynurenine pathway, without being incorporated into proteins. Kinetic analyses on dynamic acquisitions allowed precise discrimination between recurrence and radiation necrosis. The tracer also has a prognostic role, the intensity of fixation being correlated with OS (197, 198).

^18^F-fluciclovin, also called anti-[^18^F]FACBC, a new ^18^F-labeled amino acid, was tested in patients followed for gliomas in a phase II study. The authors concluded that this radiotracer was safe, and that it was able to delineate the gliomatous invasion in cases where it could go unnoticed on the MRI (199).

Another amino acid pathway is glutamine, which together with glucose, is one of the two nutrients necessary for cell proliferation and survival. A radiolabeled ^18^F-glutamine analog 4-^18^F-(2S,4R)-fluoroglutamine or ^18^F-FGln demonstrated a very high lesion/background ratio, no uptake in neuroinflammatory lesions, an absence of leakage through the permeable BBB, and a correlation between the decrease in its fixation and tumor shrinkage. It succeeded in displaying a suitable lesion/background ratio in a patient followed for a glioblastoma (200).

^11^C-(R)PK11195, selectively binds to a mitochondrial translocator protein (TSPO), which is upregulated in high-grade astrocytomas but not in healthy brain parenchyma. This tracer has theranostic potential by being a potential target for targeted treatments or for the passage of nanoparticles. TSPO was expressed predominantly by neoplastic cells, and its ligand, ^11^C-(R)PK11195, binds more strongly in high-grade gliomas than in low-grade gliomas. However, its ^11^C radiolabeling could limit its use (201). Other TSP-^18^F radiolabeled ligands, the ^18^F-DPA-714, with higher binding potential achieved visualization of gliomas in preclinical studies (202). Another ^18^F-labeled ligand investigated in patients followed for pre- or post–treatment glioblastoma, the ^18^F-GE-180, displayed a significant tumor-to-background contrast. The PET volume based on tracer uptake was larger than the volume based on MRI-enhanced tumor, thanks to tumor areas capturing the ^18^F-GE-180 even outside the areas enhanced on MRI. Broader prospective studies with histological comparison are needed to eliminate uptake related to inflammation (203).

Recent studies on ^68^Ga-radiolabeled bombesin analogs in glioma exploration are emerging. NOTA-Aca-BBN (7–14) (or ^68^Ga-BBN) tested in volunteer patients and patients followed for gliomas had no side effects and an advantageous dosimetry. The binding intensity correlated with the expression level of gastrin-releasing peptide receptor. The authors stressed the potential theranostic role of this tracer (204, 205).

^62^Cu-diacetyl-bis (N4-methylthiosemicarbazone), more simply named ^62^Cu-ATSM, could replace ^18^F-FMISO in the imaging of hypoxic metabolism in gliomas. The binding of ^62^Cu-ATSM was significantly higher in glioblastoma than in grade III glioma. Uptake correlated with the expression of the HIF-1 marker (206).

Acetate is a potential source of energy, which can be radiolabeled with ^11^C. In a study involving patients followed for gliomas, including glioblastoma, this tracer compared to ^18^F-methionine and ^18^F-FDG had a sensitivity of 90% in glioma detection (vs. 100 and 40%, respectively). Its uptake was significantly higher in high-grade gliomas than in those of low-grade (207). These results were further confirmed by another study, showing a significantly higher uptake and tumor/cortex (T/C) ratio for high-grade gliomas than for low-grade gliomas (208).

The ^18^F-labeled-2-(5-fluoro-pentyl)-2-methyl-malonic acid ([^18^F]ML10) is a useful tracer for apoptosis that characteristically highlights cells that have acquired permanent membrane depolarization. This tracer used in a small series of patients followed for glioblastoma failed to prove a correlation between changes in tracer uptake and time to progression. The authors hypothesized that this failure was secondary to a variable rate of cell growth and death between the different lesions (209).

Some authors explored integrin-specific radiopharmaceutical α_v_β_3_, ^68^Ga-BNOTA-PRGD2 (^68^Ga-PRGD2), in glioma. This tracer accumulated in integrin-rich lesions (overexpressed especially in neovessels and glioma cells) and in choroid plexuses, but not in the rest of the healthy cerebral parenchyma in a small series of patients with high-grade gliomas. SUVmax and TBRmax were both significantly correlated with tumor grade, and ^68^Ga-PRGD2 was greater than ^18^F-FDG in distinguishing low-grade glioma from high-grade glioma, even allowing discrimination between grade III glioma and grade IV glioma (210). These results confirm prior results in patients with glioblastomas, with another tracer radiolabeled with ^18^F, the Arg-Gly-Asp peptide (^18^F-galacto-RGD) (211).

^18^F-AlF-NOTA-PRGD2 (^18^F-RGD), another integrin-specific tracer, may play a predictive role in assessing early response to chemotherapy (212).

However, these tracers are only under experimentation, further larger studies are needed, including prospective clinical trials comparing these innovative tracers to the current more standard radiolabeled amino acids.

Finally, the development of PET-MRI opens up the possibility to study the various PET and MRI parameters in a single investigation and in a relatively short time. The problem of the attenuation correction is not perfectly solved and the high cost of such equipment is nevertheless a major constraint on their uptake (213).

## Synthesis and Outlook

Due to the emergence of new different therapies in the management of glioblastoma and poor prognosis of this disease, it is crucial to have reliable evaluation imaging.

PET imaging is an additional tool that should be used in conjunction with MRI, thanks to these numerous tracers and their multiple contributions. Despite an insufficient role in the differentiation between tumor recurrence and radiation injury, ^18^F-FDG still plays an important prognostic role. Regarding the use of radiolabeled amino acids, the spread of ^11^C-methionine is hampered by the need for on-site cyclotron. The ^18^F-FET and ^18^F-FDOPA are more easily accessible, thanks to their fluorine-18 radiolabeling, with excellent properties at all grades of the pathology. They display reliable performances in distinguishing post-radiation-related modifications and tumor recurrence (Table 2) and improved early assessment of tumor response during antiangiogenic treatment. These tools are currently recommended in equivocal situations only. The developement of ^68^Ga-PSMA seems very promising in the distinction between radiation injury and relapse. Its synthesis is straightforward to implement, and its availability makes it a potentially more accessible and less expensive tracer. Early distinction of tumor recurrence from a post-radiation complication at a lower cost is of paramount importance because of the soaring costs of targeted treatments. Considering the poor prognosis of this pathology, no time should be lost due to indecision. Another potential advantage of PSMA is its possible theranostic use. However, the precise place of this new tracer is yet to be defined, and powerful prospective clinical trials are needed.

**Table 2:** Main radiotracers performances in gliomas recurrence distinction and in discriminating recurrence from post-therapeutic modifications.

	**Sensitivity %**	**Specificity %**	**Positive predictive value %**	**Negative predictive value %**	**Accuracy %**
18F-FDG	43–100 (29, 42, 68, 77, 78, 100, 152)	40–100 (29, 42, 68, 77, 78, 100, 152)	80–00 (100, 152)	20–38.9 (100, 152)	60.7 (100)
11C-choline and 18F-Fcholine	73.5–92.3% (68, 77)	87.5% (68, 77)	NA	NA	NA
11C-methionine	75–91.2% (77, 78)	87.5–100 (77, 78)	NA	NA	NA
18F-FDOPA	84–100 (95, 100, 101)	62.1–100 (95, 100, 101)	89.6–100 (95, 100, 101)	63.4–100 (95, 100, 101)	78–97 (95, 100, 101)
18F-FET	84–100 (126, 128–130)	86–100 (126, 128–130)	84–100 (125, 128)	NA	85–96 (128–130)
18F-FLT	82.1 (152)	50 (152)	73.3 (152)	26.7 (152)	NA

## Author Contributions

AM and DK: conception, design, and writing all sections of the manuscript. TM, OF, and DF: provided the literature and writing sections of the manuscript.

### Conflict of Interest

The authors declare that the research was conducted in the absence of any commercial or financial relationships that could be construed as a potential conflict of interest.
